# RETRACTED: Choi, J.-Y.; Kim, S.-K. An Evolutionary Strategy for Spawning Habitat Selection by *Pseudopungtungia tenuicorpa*. *Animals* 2023, *13*, 2170

**DOI:** 10.3390/ani16111635

**Published:** 2026-05-27

**Authors:** Jong-Yun Choi, Seong-Ki Kim

**Affiliations:** 1 Department of Ecological Engineering, Pukyong National University, Busan 48513, Republic of Korea; 2 National Institute of Ecology, Seocheon-gun 33657, Republic of Korea

The Journal retracts the article “An Evolutionary Strategy for Spawning Habitat Selection by *Pseudopungtungia tenuicorpa*” [1], cited above.

Following publication, concerns were brought to the attention of the Editorial Office regarding the integrity of the data presented in the article [1].

In accordance with standard journal procedures, the Editorial Office and Editorial Board conducted an investigation, which included input from Pukyong National University and the National Institute of Ecology. The investigation determined that the integrity of the underlying data could not be confirmed. Consequently, the Editorial Board has lost confidence in the reliability of the findings and decided to retract this publication [1], as per MDPI’s retraction policy.

This retraction was approved by the Editor-in-Chief of the journal *Animals*.

The authors have been informed of this retraction but did not provide a comment on this decision.
